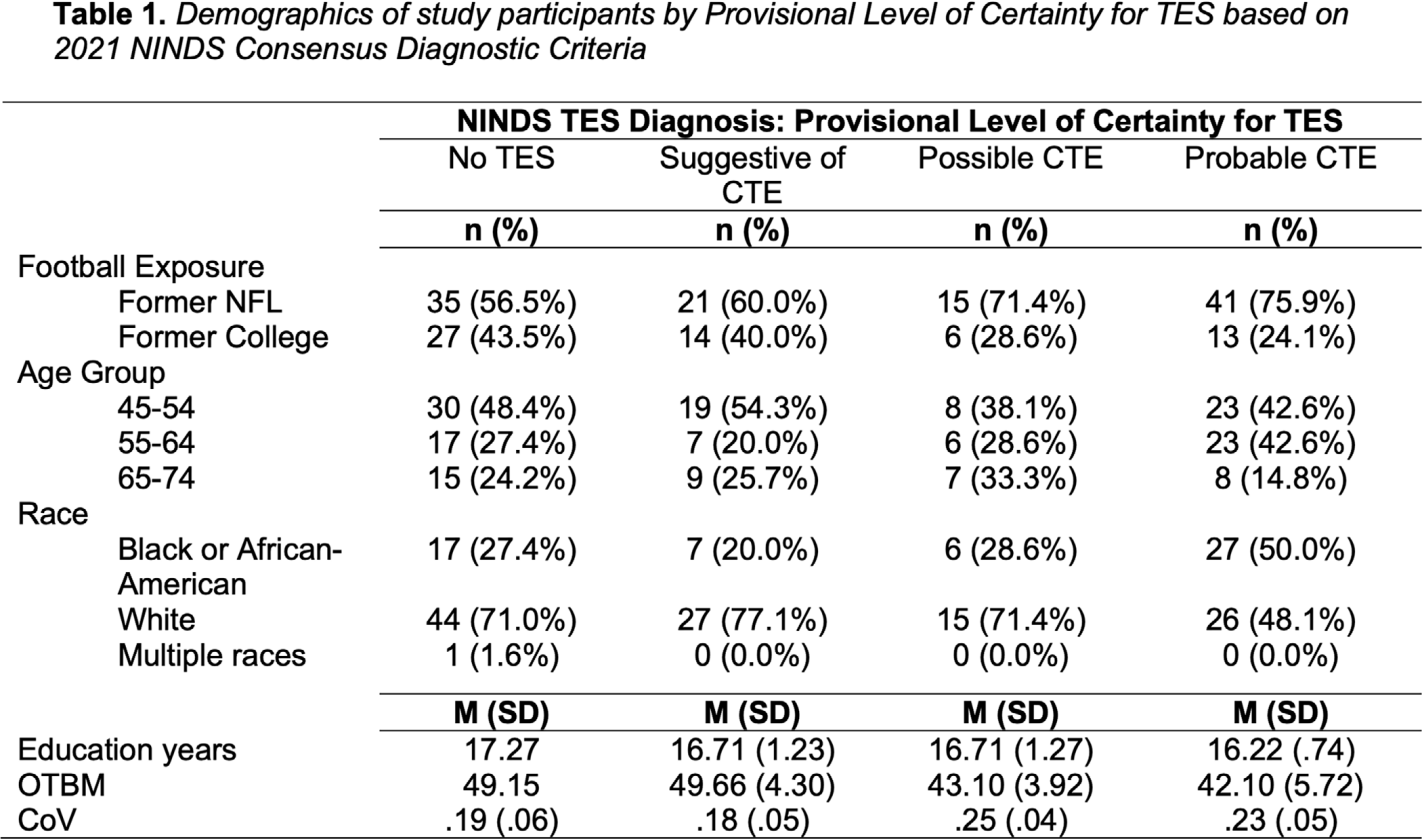# Beyond Traditional Measures: Exploring Cognitive Intraindividual Variability in Traumatic Encephalopathy Syndrome

**DOI:** 10.1002/alz.092347

**Published:** 2025-01-03

**Authors:** Caroline Altaras, Monica T. Ly, William B. Barr, Sarah Banks, Jennifer V. Wethe, Yorghos Tripodis, Charles Adler, Laura Balcer, Charles B. Bernick, Robert C. Cantu, David W. Dodick, Jesse Mez, Joseph N. Palmisano, Brett Martin, Jeffrey L. Cummings, Eric M. Reiman, Martha E. Shenton, Robert A. Stern, Douglas I Katz, Jason Weller, Yonas E. Geda, Michael L. Alosco

**Affiliations:** ^1^ Boston University Chobanian & Avedisian School of Medicine, Boston, MA USA; ^2^ Boston University Alzheimer’s Disease Research Center, Boston, MA USA; ^3^ NYU Langone Health, New York City, NY USA; ^4^ University of California, San Diego, La Jolla, CA USA; ^5^ Mayo Clinic, Scottsdale, AZ USA; ^6^ Lou Ruvo Center for Brain Health, Cleveland Clinic, Las Vegas, NV USA; ^7^ University of Washington, Seattle, WA USA; ^8^ Boston University Chronic Traumatic Encephalopathy Center, Boston University Chobanian & Avedisian School of Medicine, Boston, MA USA; ^9^ Chambers‐Grundy Center for Transformative Neuroscience, Department of Brain Health, School of Integrated Health Sciences, University of Nevada Las Vegas, Las Vegas, NV USA; ^10^ Banner Alzheimer’s Institute, Phoenix, AZ USA; ^11^ Translational Genomics Research Institute, Phoenix, AZ USA; ^12^ University of Arizona, Phoenix, AZ USA; ^13^ Arizona Alzheimer’s Consortium, Phoenix, AZ USA; ^14^ Arizona State University, Phoenix, AZ USA; ^15^ Brigham and Women’s Hospital, Boston, MA USA; ^16^ Boston University, Boston, MA USA; ^17^ Barrow Neurological Institute, Phoenix, AZ USA

## Abstract

**Background:**

Traumatic encephalopathy syndrome (TES) is the proposed clinical syndrome of chronic traumatic encephalopathy (CTE), a neurodegenerative disease associated with repetitive head impacts in contact/collision sports. A core clinical feature of TES is cognitive impairment, particularly in memory and executive functions. Cognitive intraindividual variability (IIV) is the extent of variability in neuropsychological test performance (i.e., dispersion of test scores) and is believed to be an indicator of disruption in executive control, linked to altered fronto‐subcortical circuits. Here, we examined cognitive IIV as a method to detect subtle executive dysfunction in TES that may not be captured by traditional neuropsychological measures.

**Method:**

The sample included former American football players (120 professional, 60 college); participants were males, aged 45‐74. Participants completed a baseline neuropsychological test battery (n = 8 excluded for suboptimal performance validity). A multidisciplinary diagnostic consensus conference classified participants using the 2021 NINDS Consensus Diagnostic Criteria for TES (see Table‐1): (1) No‐TES (n = 62); (2) TES‐CTE Suggestive (n = 35); (3) TES‐CTE Possible (n = 21); (4) TES‐CTE Probable (n = 54). TES‐CTE Possible and Probable were combined. The coefficient of variation (CoV) was used to measure cognitive IIV (CoV = iSD/iOTBM, iOTBM = average T‐score across neuropsychological tests; iSD = standard deviation of iOTBM). A higher CoV indicates greater score dispersion. To avoid circularity, memory and executive tests used for classifying TES groups were intentionally excluded from the CoV calculation. Analysis of covariance compared the TES groups on CoV, controlling for age, education, and race.

**Result:**

Results showed a significant difference in CoV between TES groups, F(2, 165) = 8.20 p<.001, partial η2 = .09. The TES‐CTE Possible/Probable group had the highest CoV (M = .226, SE = .007), compared with the TES‐no (M = .192, SE = .008, p<.001) and TES‐CTE Suggestive groups (M = .178, SE = .011, p<.001). CoV did not significantly differ between the TES‐no and TES‐CTE suggestive groups (Mdiff = 0.014, SE = .013, p = .293).

**Conclusion:**

The TES‐CTE possible/probable group had the highest cognitive IIV with ∼23% dispersion (vs. ∼18‐19% in TES‐no and TES‐CTE suggestive groups), suggesting greater cognitive variability in those with the greatest likelihood for having underlying CTE (based on TES criteria). This finding emphasizes the potential utility of exploring cognitive IIV as a method for detecting nuanced executive dysfunction in TES.